# Multidimensional biological activities of resveratrol and its prospects and challenges in the health field

**DOI:** 10.3389/fnut.2024.1408651

**Published:** 2024-06-12

**Authors:** Xi Yu, Yuanqiang Jia, Feiyue Ren

**Affiliations:** School of Food and Health, Beijing Advanced Innovation Center for Food Nutrition and Human Health, Beijing Engineering, and Technology Research Center of Food Additives, Beijing Technology and Business University (BTBU), Beijing, China

**Keywords:** resveratrol, biological activity, bioavailability, clinical research, limitation

## Abstract

Resveratrol (RES) is a naturally occurring polyphenolic compound. Recent studies have identified multiple potential health benefits of RES, including antioxidant, anti-inflammatory, anti-obesity, anticancer, anti-diabetic, cardiovascular, and neuroprotective properties. The objective of this review is to summarize and analyze the studies on the biological activities of RES in disease prevention and treatment, as well as its metabolism and bioavailability. It also discusses the challenges in its clinical application and future research directions. RES exhibits significant potential in the prevention and treatment of many diseases. The future direction of RES research should focus on improving its bioavailability, conducting more clinical trials to determine its effectiveness in humans, and investigating its mechanism of action. Once these challenges have been overcome, RES is expected to become an effective health intervention.

## 1 Introduction

**Figure 1:**
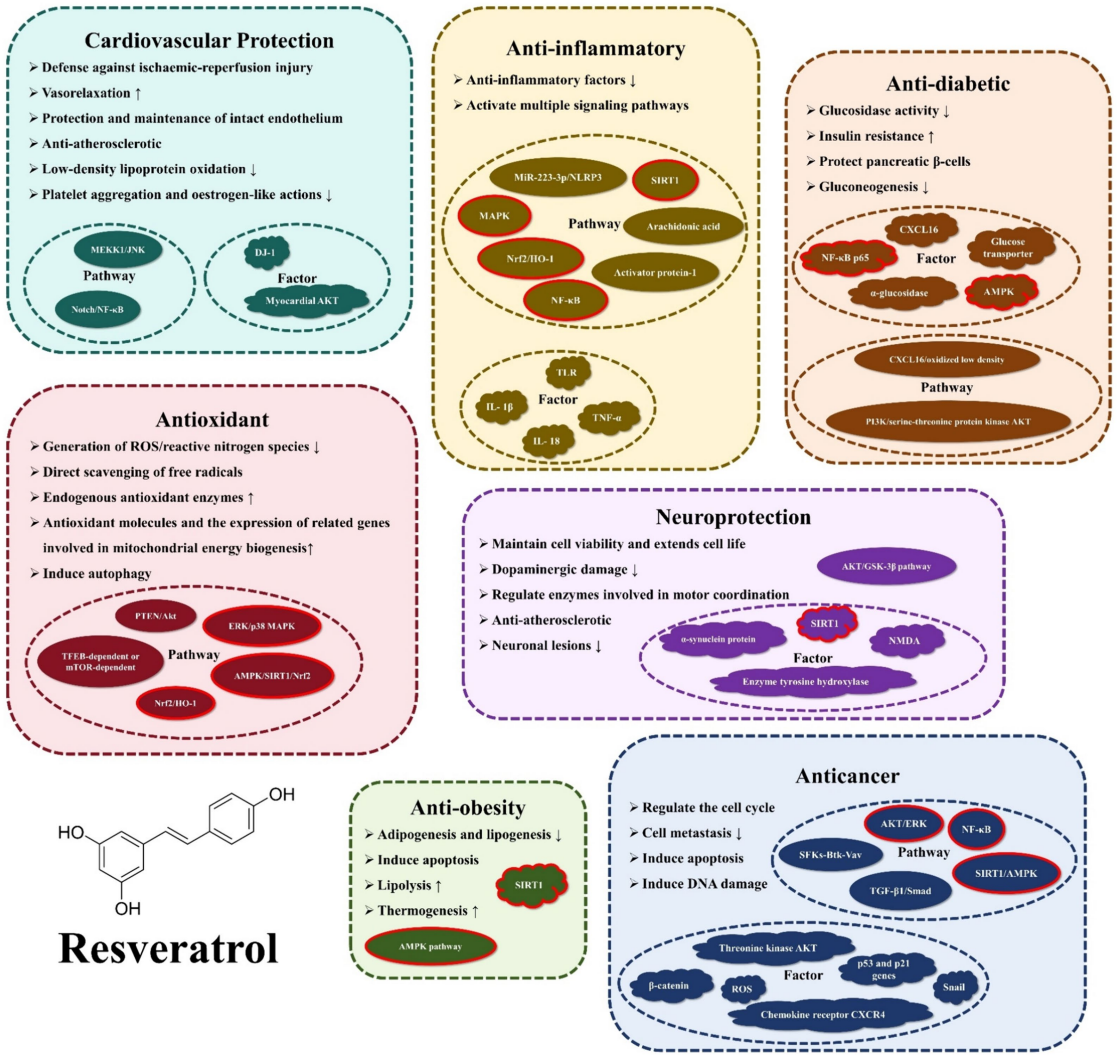
Functions and mechanisms of action of resveratrol.

RES (3,4′,5-trihydroxy-trans-stilbene) is a polyphenolic compound that contains three hydroxyl groups (-OH) (1). And it is naturally present in nuts, fruits, and their processed products. Michio Takaoka first identified RES in 1939, extracting it from the root of the white hellebore (2). Early studies focused on its chemical structure and botanical properties, but little on physiology. However, in 1992, Siemann and Creasy revealed RES’s lipid-lowering properties, sparking scientific curiosity in the potential physiological benefits of this natural stilbene (3). In 1997, Meishiang Jang et al. demonstrated the potent anticancer effects of RES during tumor initiation, promotion, and progression stages (4). Further research has unveiled a myriad of additional functions of RES, including its antioxidant, anti-inflammatory, anti-obesity, anti-diabetic, cardiovascular protective, and neuroprotective effects, among others. Figure 1 illustrates the biological activities and mechanisms of action of the RES that will be presented in this review.

In biology, RES is a phytotoxin that is produced when plants are injured, which can be used to defend against stress. Under harsh conditions, plants may increase RES biosynthesis (5). Currently, the methods of synthesizing RES can be classified into plant extraction, biosynthesis and chemical synthesis. Extraction from plants is limited by factors such as low yield or growth habit, and the extraction efficiency of this method is low. Nevertheless, there are still a few companies engaged in the production of trans-resveratrol with varying purities derived from the root extract of *Polygonum multiflorum* (6). As the field of biosynthetic methods continues to advance, it offers the advantage of high productivity and low cost. As a result, it has become the dominant method for industrial synthesis of RES. The microorganisms used for RES production are mainly endophytic fungi. In one study, 13 endophytic fungal isolates were examined and *Alternaria alternata* was found to produce 206 μg/L of RES with the highest yield (7). In contrast, chemical synthesis methods require strict conditions and precise control, which renders them more commonly used in laboratory synthesis (8).

The purpose of this review is to provide an overview of the biological activities of RES and its potential applications in disease prevention and treatment. The objective of this research is to provide insights into future research directions and strategies for overcoming existing challenges in order to fully realize the potential of RES in medicine and health. We synthesize and analyze current research results to achieve this objective.

## 2 Metabolism and bioavailability of RES

### 2.1 Absorption, metabolism and excretion

RES has an oral absorption rate of up to 70%. It crosses the intestinal cell parietal membrane through either passive diffusion or carrier-mediated transport mechanisms. Once absorbed, RES is rapidly and extensively metabolized in the liver and intestine, predominantly via glucuronidation and sulfonation processes (9). The compound has a plasma half-life of approximately 9.2 ± 0.6 h. However, the bioavailability of this substance is notably low, less than 1%, with an estimated 20%—30% of RES unaccounted for in urine or feces (10).

**Figure 2:**
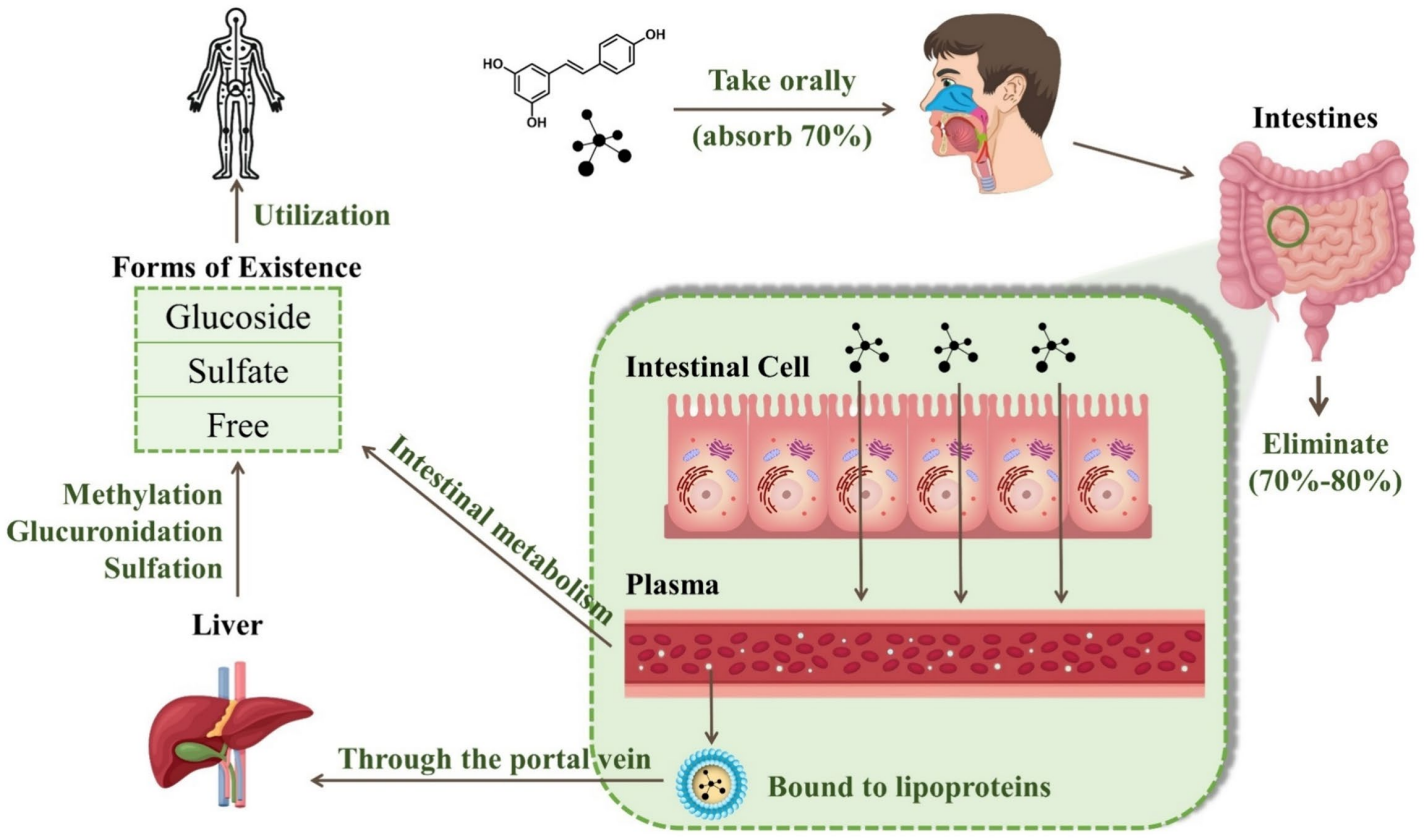
Digestive metabolism of resveratrol in the human body.

RES can exist in one of three forms: glucoside, sulfate, or free. During intestinal metabolism, RES associates with ATP-dependent binding cassette membrane transport proteins, resulting in the production of sulfate and glucuronide derivatives. These derivatives are then released into the bloodstream in their free form (11). Due to its lipophilic properties, more than 90% of free trans-RES is bound to human plasma lipoproteins. After being absorbed through the portal vein, the polyphenol metabolites undergo further methylation, glucuronidation, or sulfation in the liver. This is known as Phase II metabolism, which is primarily characterized by enzyme-driven conjugation reactions that produce a diverse array of metabolites (12). Notably, phase II metabolites vary between species, with the primary conjugate produced by RES in humans being sulfate, whereas the preferred conjugate in animals such as pigs and rats is glucuronide. Specifically, RES engages in conjugation with sulfate and glucuronate via the catalysis of sulfotransferase and uridine-5′-diphosphate-glucuronate uridylyltransferase, respectively (13). These metabolites then permeate through the systemic circulation and reach the target tissues and cells. Both RES and its unused metabolites can be redirected to the small intestine via bile or excreted through urine (14). There are five main forms of RES in urine: resveratrol monosulfate, two isomeric forms of resveratrol monoglucuronide, dihydroresveratrol monosulfate, and dihydroresveratrol monoglucuronide (15). Figure 2 illustrates the changes in RES in the human body through oral administration.

### 2.2 Factors affecting bioavailability

**Figure 3:**
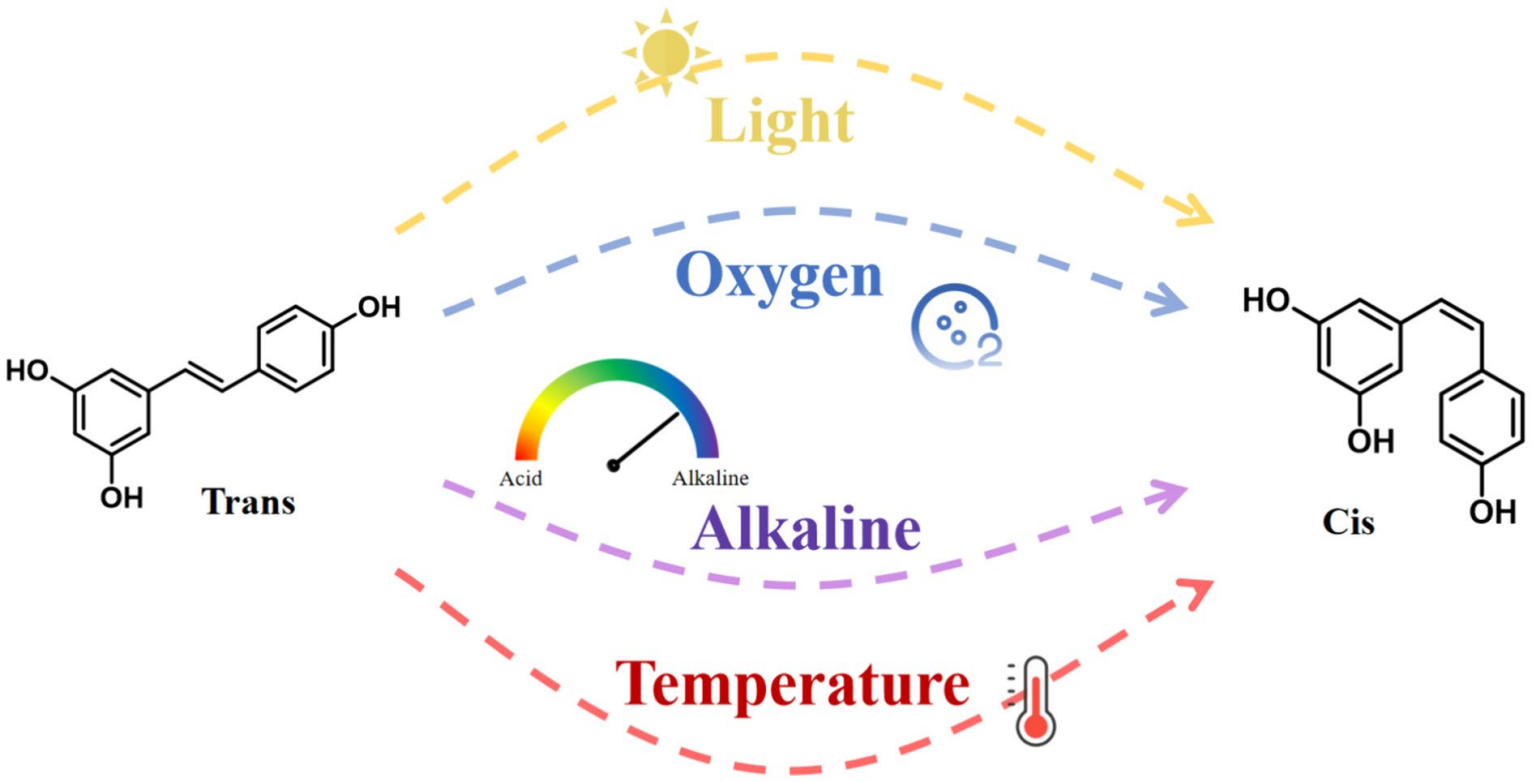
Cis- and trans-structures of resveratrol and factors affecting their conversion.

RES has poor water solubility and is chemically unstable. It degrades when exposed to light, oxygen, extreme temperatures, and alkaline conditions (Figure 3). These environmental stressors cause isomerization and autoxidation, converting RES from its trans to its cis configuration (16). Studies suggest that the trans configuration of RES is more biologically active than its cis counterpart (17). Importantly, the glucuronidation process is 5 to 10 times faster in the cis-form, resulting in lower bioavailability of the cis-form than in the trans-form (18). Additionally, the bioavailability of RES also varies depending on an individual’s physical condition. In the human body, albumin serves as a potential reservoir for RES, binding to it and affecting its distribution and bioavailability (15).

Some external treatments can also affect the bioavailability of RES. The utilization of micro- and nano-delivery systems can improve the surface-to-volume ratio, increase the solubility and stability of bioactive compounds, customize their release. The most important thing is that the bioavailability of RES can be improved in this way (19). For example, the integration of RES with α-tocopherol has been demonstrated to improve the chemical stability of α-tocopherol during storage and *in vitro* digestion. Incorporating acacia gum and low concentrations of pectin has been observed to enhance the bioavailability of both α-tocopherol and RES (20). This enhancement may be due to increased protective effects during digestion, resulting in more stable RES existing by the conclusion of the digestive process. Besides, polymer particles are currently one of the most commonly used micro- and nanostructures in the food industry. It has been found that particles of whey protein concentrate or sodium caseinate produced by spray drying techniques, which encapsulate 70–80% of RES, can enhance digestive stability and bioavailability by 47 and 23%, respectively (21). In addition, combinations or synergies are a good way to increase the bioavailability of RES. The scientific literature contains numerous instances of combination or synergistic effects. Further investigation is warranted into the differential effects produced by RES, both in isolation and when combined with other compounds. For instance, quercetin has been shown to inhibit the *in vivo* sulfation of RES, which could potentially increase RES’s bioavailability within the body (22).

In summary, a combination of factors contributes to its bioavailability, including its nature, the environment, drug interactions, and other factors. Understanding these factors can assist in the utilization of RES.

## 3 The bioactivities of RES

RES has high biological activity and is capable of antioxidant, anti-inflammatory, anti-diabetic, anti-obesity, anti-cancer, cardiovascular and neurological protection. For these properties, we have summarized some studies and analyzed them, what is changed by the action of RES and what are the possible applications of such changes, as shown in Table 1.

**Table 1:** Treatment perspectives of resveratrol.

Perspective	Biomarker changes	Application prospects	References
Antioxidant	HO-1, glutathione, glutathione peroxidase, and superoxide dismutase ↑ MDA, TNF-α, or inflammatory cells ↓	Alleviate oxidative DNA damage in kidney and liver tissue caused by sepsis	(23)
	Activated AMPK/p38/Nrf2 signaling pathway	Improve myocardial ischemia–reperfusion injuries in diabetic patients	(24)
	TNF-α and MMP-9 ↓	Alleviate symptoms of the autism spectrum disorders	(25)
Inflammation	TLR 2, TLR 4, and proinflammatory cytokines ↑	Accelerate the onset of the inflammatory response	(26)
	TLR activity ↓ and proinflammatory factors ↓ TLR4 levels in brain cells ↑	Reduce central nervous system inflammation	(27)
	Modulated the miR-223-3p/NLRP3 pathway	Protecting the neurons	(28)
	IL-8 ↓ and activated the Nrf2/HO-1 signaling pathway	Alleviate *H. pylori*-induced gastric inflammation	(29)
	IL-1β and TNF-α ↓ Inflammatory markers ↑	Mitigate both the inflammatory condition and oxidative stress	(30)
	Activated the SIRT1 signaling pathway	Alleviate manganese-induced oxidative stress and inflammation	(31)
Anti-diabetic	CXCL16 and the NF-κВ p65 ↓	Beneficial for type I diabetes	(32)
	Blood glucose, triglycerides, and body weight ↓ Insulin sensitivity ↑	Beneficial for type II diabetes	(33)
	Activated PI3K/AKT signaling pathway and inhibit FoxO1	Hypoglycemic	(34)
	Inhibited α-glucosidase	Improve glycemic control	(35)
	AMPK activity ↑	Facilitate glucose transport	(36)
Anti-obesity	Exercise performance and insulin sensitivity ↑	Anti-obesity	(13)
	No significant impact on weight loss	–	(37)
	Body weight, body mass index, fat mass, and leptin levels ↓	Have an effect on obesity in the body	(38)
	In liver and skeletal muscle tissues: lipid oxidation ↑	Reduce lipid accumulation	(39)
	In white adipose tissue: adipogenesis and/or *de novo* lipogenesis ↓	Reduce fat storage, and enhance the browning process	
	In brown adipose tissue: thermogenic capacity ↑	Anti-obesity	
	Activated oxidation, inhibit fat accumulation, and promote lipolysis	Anti-obesity	(40)
	SIRT1 protein, AMPK activity and mitochondrial biogenesis ↑	Modulate cellular energy utilization and aid muscle regeneration in obese individuals	(31)
Anticancer	Inhibited TNF-receptor associated factor 6	Potential to inhibit prostate cancer	(41)
	Inhibit glucose fermentation and promote respiration	Slow down the growth of PC3 cells	(42)
	Low concentrations: the ratio of G0/G1 populations ↓ High concentrations: the ratio of G0/G1 populations ↑	Lead to cell cycle arrest	(43)
	Inhibited threonine kinase AKT	Induce apoptosis in cancer cells	(44)
	Superoxide free radicals and activating caspase-3 ↑	Provoke apoptosis	(45)
	Activated NADPH oxidase, ROS, p53 and p21 gene ↑	Cause DNA damage in cancer cells	(46)
	Targeted the SFKs-Btk-Vav pathway	Inhibit cancer cell metastasis	(47)
	Activated cytokine signal transducer 1	Inhibit cancer cell proliferation	(48)
	Modulated SIRT1/AMPK signaling pathway	Regulate physiological processes	(49)
Cardiovascular protection	Modulated of the Notch/NF-κB signaling pathway	Protect the heart from aging-related dysfunction	(50)
	Regulated DJ-1 protein and the MEKK1/JNK signaling pathway	Inhibit autophagy	(51)
	Reverse impaired cardiac diastolic and systolic functions Improve myocardial structural disorders and fibrosis Inhibit cardiomyocyte apoptosis	Treat diabetic cardiomyopathy	(52)
	Myocardial AKT ↑	Reduce myocardial injury	(53)
Neuroprotection	MPP + -induced cytotoxicity ↓	Prevent neuronal damage	(54)
	Regulated calcium influx triggered by NMDA receptor activation	Neuroprotective agent	(55)
	Dendrite length and spine density of pyramidal neurons ↑	Protect and enhance neural architecture	(56)

### 3.1 Antioxidant

There is a balance between oxidant production and antioxidant defense system function under normal circumstances. However, when the production of catalytic oxides exceeds the system’s capacity to neutralize and eliminate them, this balance is disrupted, leading to oxidative stress. This imbalance may cause damage to biological systems. Reactive pro-oxidative molecules, such as reactive oxygen species (ROS) and reactive nitrogen species, are generated during metabolic processes within living cells. Heme oxygenase is an intracellular enzyme that catalyzes the degradation of heme into products with antioxidant properties. RES has been demonstrated to reduce the accumulation of ROS and increase the expression of heme oxygenase-1 (HO-1). Besides, atherosclerosis is a leading cause of mortality worldwide. The production of bilirubin and carbon monoxide (CO) by HO-1 exhibits protective properties against atherosclerotic diseases (57). Furthermore, RES has been shown to increase levels of glutathione, superoxide dismutase, and glutathione peroxidase, while reducing the expression of malondialdehyde, TNF-α, and inflammatory cells. This demonstrates its potential to alleviate oxidative DNA damage in kidney and liver tissue caused by sepsis (23).

The results of numerous studies have demonstrated the broad therapeutic potential of RES for diseases caused by oxidative stress. The Adenosine 5′ monophosphate-activated protein kinase (AMPK)/p38/Nrf2 signaling pathway is an endogenous antioxidant stress response that provides protection from various stressors. The ability of RES to activate this signaling pathway highlights its potential in mitigating oxidative stress and improving myocardial ischemia–reperfusion injuries in diabetic patients (24). Oxidative stress is an important pathological process secondary to spinal cord injury, and RES can inhibit oxidative stress by activating the Nrf2/HO-1 signaling pathway (58). Oxidative stress is one of the major factors contributing to wound healing difficulties in diabetic patients, and RES activates Sirtuin 1 (SIRT1) to restore hyperglycemia-induced endothelial dysfunction and angiogenic disorders, thereby promoting wound healing (59). Additionally, autism spectrum disorders have been the focus of research due to their potential link to oxidative stress and mitochondrial dysfunction. Studies suggest that RES may alleviate the core and associated symptoms of the autism spectrum disorders rat phenotype by reducing oxidative stress, decreasing the expression of tumor necrosis factor-α (TNF-α) and MMP-9, and addressing mitochondrial dysfunction (25).

The resveratrol (RES) can mitigate oxidative stress through multiple mechanisms: (a) decreasing the generation of ROS/reactive nitrogen species; (b) directly scavenging free radicals; (c) enhancing endogenous antioxidant enzymes; (d) promoting antioxidant molecules and the expression of related genes involved in mitochondrial energy biogenesis, mainly through AMPK/SIRT1/Nrf2, ERK/p38 Mitogen-activated protein kinase (MAPK), and PTEN/Akt signaling pathways; and (e) inducing autophagy via mTOR-dependent or TFEB-dependent pathway (60, 61).

### 3.2 Anti-inflammatory

Inflammation and oxidative stress play a critical role in causing several diseases. ROS have an important role in the upregulation of the expression of Toll-like receptor (TLR) 2 and TLR4, thereby accelerating the onset of the inflammatory response. Additionally, ROS can increase the expression of proinflammatory cytokines, further promoting inflammation (26). RES inhibits TLR activity and reduces the expression of proinflammatory factors. Studies involving nano-RES have highlighted its potential to increase TLR4 levels in brain cells and suppress the activation of inflammatory mediators. This evidence underscores the anti-inflammatory properties of RES, particularly in the context of central nervous system inflammation (27).

The neuroprotective effects of RES extend to safeguarding cortical neurons from inflammatory insults and cell death by modulating the miR-223-3p/NLRP3 pathway. This modulation prevents the activation of caspase-1 and the subsequent processing of interleukin (IL)-1β and IL-18 in neurons and BV-2 cells (28). Furthermore, *Helicobacter pylori* infection, which is a primary cause of inflammation in the stomach and duodenum, is linked to the overexpression of IL-8 and an increase in the expression of inducible nitric oxide synthase (62). Inducible nitric oxide synthase is an enzyme produced in response to inflammation and has been implicated in the development of *H. pylori*-induced gastritis. Pylori-induced gastritis (63). RES has been shown to effectively alleviate *H. pylori*-induced gastric inflammation by inhibiting IL-8, a key proinflammatory mediator, and activating the Nrf2/HO-1 signaling pathway to reduce inducible nitric oxide synthase expression (29).

The Goto-Kakizaki (GK) rat is a non-obese model for type II diabetes. In diabetic rats, a 10-week treatment with RES inhibited the rise of inflammatory markers in skeletal muscle and decreased the concentrations of IL-1β and TNF-α measured in the livers of GK rats. These results are an indication that RES has therapeutic effects that can alleviate both the oxidative stress and inflammatory state in this model (30). Moreover, RES has been demonstrated to alleviate manganese-induced oxidative stress and inflammation in the nervous system by activating the SIRT1 signaling pathway (31). These studies collectively emphasize the potent anti-inflammatory and antioxidant capabilities of RES, highlighting its therapeutic potential in conditions characterized by inflammation and oxidative stress.

The mechanism of anti-inflammatory action of RES can be divided into two parts, the most notable manifestation of which lies in the inhibition of anti-inflammatory factors, such as TLR, IL-1β, IL-18, TNF-α, and so on. In addition, RES can also act on multiple pathways. A considerable body of research has demonstrated that resveratrol exerts its anti-inflammatory effects through a multitude of signaling pathways, including the arachidonic acid pathway, nuclear factor kappa B (NF-κB), MAPK, and activator protein-1 signal pathways (64). Each of these pathways can positively influence anti-inflammation in the presence of RES.

### 3.3 Anti-diabetic

RES has shown promise in managing diabetes, with potential therapeutic effects in both type I and type II diabetes through various mechanisms. Studies have shown that RES can suppress the expression of islet CXC chemokine ligand 16 (CXCL16) and the NF-κВ p65 in type I diabetes (32). This suggests a pathway through which RES may exert beneficial effects on type I diabetes. What′s more, RES has a protective effect on pancreatic β-cells in type I diabetes, potentially reducing β-cell expression by modulating the CXCL16/oxidized low density lipoprotein pathway (65).

The administration of RES in high-fat diet-induced diabetic mice has shown significant improvements in metabolic parameters. These improvements include reductions in blood glucose, triglycerides, and body weight, as well as an enhancement in insulin sensitivity (33). These effects collectively contribute to better glycemic control, highlighting the potential of RES as an anti-diabetic agent in type II diabetes. The mechanism of action of RES in type II diabetes also involves inhibiting gluconeogenesis, a process that contributes to elevated blood glucose levels in the fasting state. RES has been found to specifically inhibit the action of dapagliflozin, a drug known to induce gluconeogenesis, by activating the Phosphatidylinositol 3-kinase (PI3K)/serine–threonine protein kinase AKT signaling pathway. This activation results in the inhibition of factor O1, a transcription factor that stimulates gluconeogenesis, thereby achieving a hypoglycemic effect (34). In an *in vitro* cellular experiment, a significant action of RES is its ability to inhibit α-glucosidase, an enzyme involved in the breakdown of carbohydrates in the small intestine. By inhibiting α-glucosidase, RES reduces postprandial blood glucose spikes, contributing to improved glycemic control (35). Additionally, in another *in vitro* cellular experiment a RES intervention can alleviate insulin resistance (IR) and enhance the activation of AMPK (66). As an important enzyme in energy regulation, activation of AMPK by RES enhances glucose uptake in muscle and other cells, improves insulin sensitivity, and facilitates glucose transport by regulating glucose transporter 1 and glucose transporter 4 (36).

RES can exert its anti-diabetic effects through the coordination of four mechanisms: inhibiting glucosidase activity, improving IR, protecting pancreatic β-cells, and inhibiting gluconeogenesis via signaling pathways such as PI3K/serine–threonine protein kinase AKT. Furthermore, diabetes is often closely associated with oxidative stress and inflammation, and RES can achieve its anti-diabetic effects through its antioxidant and anti-inflammatory properties.

### 3.4 Anti-obesity

Obesity is a significant global health concern that contributes to various chronic conditions. Recent evidence highlights the beneficial impact of RES in combating obesity through various mechanisms, such as adipogenesis, apoptosis, lipogenesis, lipolysis, and thermogenesis (67).

SIRT1 is a molecular target of RES and plays a crucial role in regulating mitochondrial homeostasis and cellular energy metabolism. RES significantly upregulates SIRT1 protein levels, thereby modulating cellular energy utilization. Additionally, RES positively influences the AMPK pathway, which is crucial for regulating adipose tissue lipogenesis. Supplementation of RES in mouse models has demonstrated slowed fat regeneration in muscle, enhanced AMPK activity, and increased mitochondrial biogenesis. These findings suggest that it may be possible to aid muscle regeneration in obese individuals through such supplementation (31). Furthermore, RES enhances exercise performance and insulin sensitivity (13). However, the effectiveness of RES supplementation in reducing obesity has yielded mixed results. While one meta-analysis reported no significant impact on weight loss (37). In contrast, another study, also a meta-analysis, highlighted substantial reductions in body weight, body mass index, fat mass, and leptin levels following RES supplementation (38). These contrasting findings indicate the need for further research to determine the conditions under which RES may exhibit significant anti-obesity effects.

RES has tissue-specific effects that combat obesity. In liver and skeletal muscle tissues, it promotes lipid oxidation, which reduces lipid accumulation. In white adipose tissue, RES decreases adipogenesis and/or *de novo* lipogenesis, thereby reducing fat storage, and enhances the browning process, which is associated with increased energy expenditure. In brown adipose tissue, it amplifies thermogenic capacity, further contributing to its anti-obesity effects (39). In summary, RES achieves its anti-obesity effects by activating oxidation, inhibiting fat accumulation, and promoting lipolysis (40).

**Figure 4:**
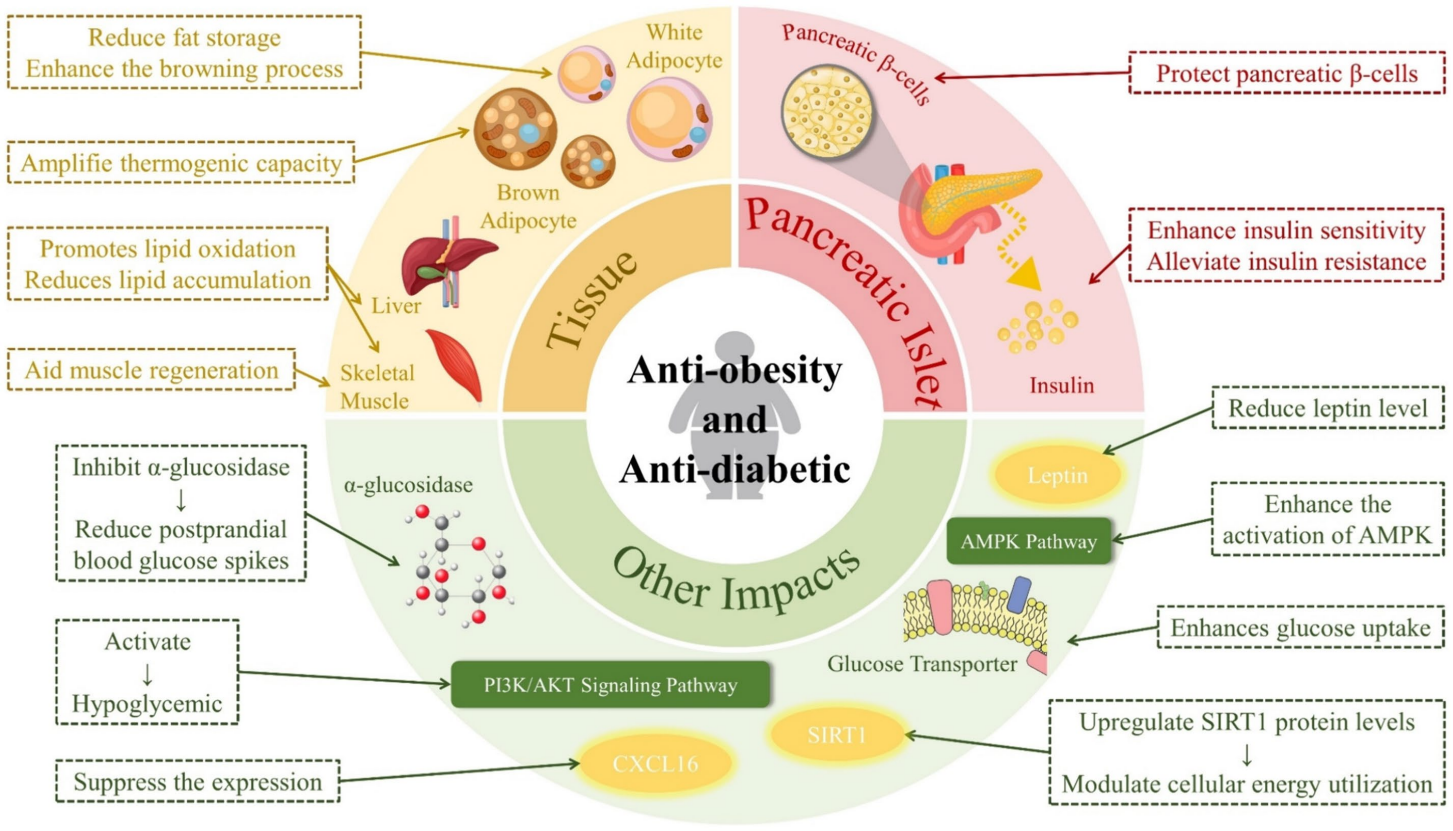
Effects of resveratrol on diabetes and obesity.

In fact, there is a strong link between obesity and diabetes, and obesity is one of the causes of type II diabetes (68). RES has an effect on both obesity and diabetes, as shown in Figure 4.

### 3.5 Anticancer

**Figure 5:**
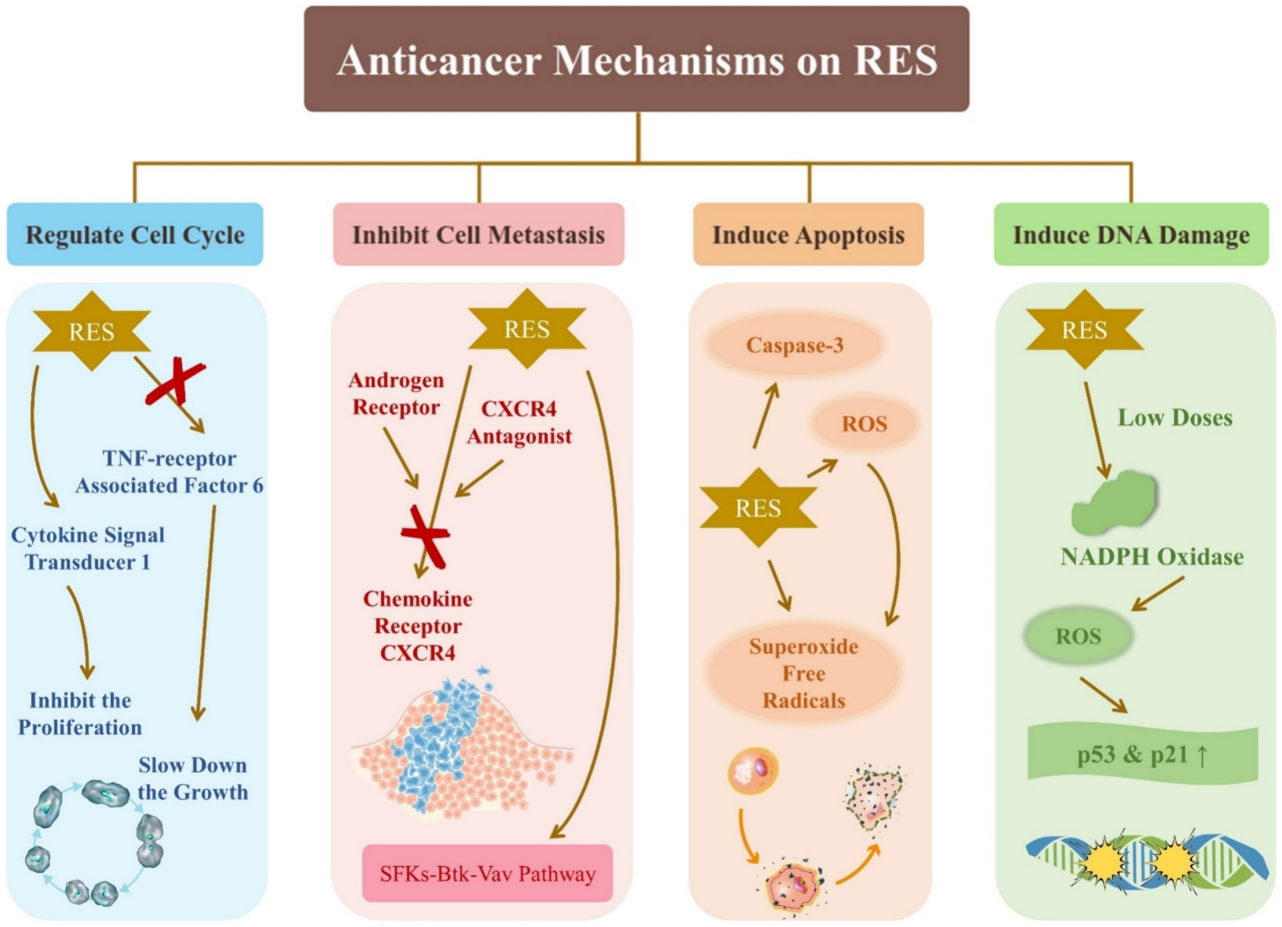
Anticancer mechanisms on resveratrol.

RES has significant potential as an anticancer agent. *In vitro* cellular experiments have been used predominantly in the study of the effect of RES on cancer, and some of the findings are summarized as follows. As shown in Figure 5, the effects of RES on cancer include regulating the cell cycle, inhibiting cell metastasis, inducing apoptosis, and inducing DNA damage.

RES acts on several fronts against cancer cell proliferation and migration by targeting lysosomal degradative pathways and inhibiting TNF receptor-associated factor 6, a known mediator of prostate cancer (PCa) cell proliferation and migration (41). In addition, RES inhibits glucose fermentation and promotes respiration, which slows down the growth of PC3 cells, even under hypoxic conditions that typically promote cancer cell survival and proliferation (42). RES is a key factor in regulating the behavior of PCa cells, indicating its ability to significantly influence the cell cycle. The androgen receptor can affect PCa treatment. Cancer metastasis often involves up-regulation of chemokine receptor CXCR4. The combination of RES with androgen receptor and CXCR4 antagonists has demonstrated efficacy in inhibiting PCa metastasis (69). It has been shown that RES can lead to cell cycle arrest: low concentrations of Res significantly decreased the ratio of G0/G1 populations, whereas high concentrations of Res increased this ratio (43).

Threonine kinase AKT, known for inhibiting apoptosis, is inactivated by RES-induced ROS upregulation, which induces apoptosis in cancer cells (44). As most tumor cells experience higher levels of oxidative stress than normal cells, they are more vulnerable to external ROS inducers. Modulating ROS levels has been shown to be a viable strategy for inducing cell death in tumor cells (70). For example, RES has been found to provoke apoptosis in colon cancer HT-29 cells by generating superoxide free radicals and activating caspase-3 (45). In addition, low doses of RES can activate NADPH oxidase to produce ROS, which leads to the upregulation of the p53 and p21 genes and subsequent DNA damage (46).

RES has been shown to inhibit cancer cell metastasis by targeting the SFKs-Btk-Vav pathway (47). Additionally, it can activate cytokine signal transducer 1 to block the signal transducer and activator of transcription 3 signaling pathway, thereby inhibiting the proliferation of head and neck squamous cancer cells (48). It also prevents the TGF-β1/Smad pathway from becoming activated, demonstrating its potential as an anticancer agent (71). Studies have shown that proanthocyanidins and RES may have synergistic anticancer effects by inhibiting the AKT and ERK signaling pathways and enhancing the NF-κB signaling pathway (72). The regulation of physiological processes, such as cell proliferation, differentiation, autophagy, apoptosis, protein synthesis and degradation, involves the SIRT1/AMPK signaling pathway (49). RES can induce autophagy in cancer cells by modulating this pathway, promoting cell apoptosis and offering a promising avenue for cancer therapy.

The main molecular mechanisms of anticancer effects of RES include the following six aspects: (a) RES downregulates the expression of β-catenin and blocks β-catenin nuclear translocation through perturbation of the long noncoding RNA MALAT1; (b) it suppresses TGF-β/Smad-induced epithelial-mesenchymal transition and transcription factor Snail; (c) it lowers the expressions of IKK-induced TNF-β, leading to the inhibition of cancer cell proliferation through deactivation of NF-ḳB; (d) it inhibits p-PI3K/p-AKT-mediated FOXO3a nuclear accumulation; (e) it suppresses the phosphorylation of Src-STAT3 and induces apoptosis of cancer cells; (f) it inhibits AKT/MAPK-induced HIF-1α activation and accelerates the degradation of the HIF-1α protein via ubiquitination (73).

### 3.6 Cardiovascular protection

Cardiovascular disease is a significant global health concern and is the leading cause of mortality among the elderly population. Oxidative stress and inflammation are common contributors to cardiovascular disease, and RES has been identified as a potent inhibitor of oxidative stress and inflammation in cardiac tissues. This effect is achieved through modulation of the Notch/NF-κB signaling pathway, offering protection against cardiac dysfunction associated with aging (50).

Recent studies have emphasized the role of RES in modulating autophagy, particularly in the context of myocardial IR injury. In cardiac cells treated with ischemia–reperfusion, DJ-1, a multifunctional protein that participates in transcription regulation and antioxidant defense, has been shown to provide significant protection. RES has been shown to inhibit autophagy through the regulation of the DJ-1 protein and its impact on the MEKK1/JNK signaling pathway (51). In a study of diabetic cardiomyopathy, RES was found to reverse impaired cardiac diastolic and systolic functions, improve myocardial structural disorders and fibrosis, and inhibit cardiomyocyte apoptosis in diseased rats. The potential of RES in the treatment of diabetic cardiomyopathy was demonstrated (52). Tissue hypoxia, often triggered by carbon monoxide exposure, is a notable factor contributing to cardiovascular complications. In this context, RES has gained attention for its cardioprotective properties. Research involving rat models has demonstrated that RES can mitigate cardiac toxicity induced by carbon monoxide. Different dosages of RES (1, 5, and 10 mg/kg) have been shown to significantly reduce myocardial injury while enhancing the expression of myocardial AKT, a key protein involved in cellular survival pathways (53).

RES protects the cardiovascular system by mechanisms that include defense against ischaemic-reperfusion injury, promotion of vasorelaxation, protection and maintenance of intact endothelium, anti-atherosclerotic properties, inhibition of low-density lipoprotein oxidation, suppression of platelet aggregation and oestrogen-like actions (74). In addition, the antioxidant and anti-inflammatory properties of RES play a major role in cardiovascular protection.

### 3.7 Neuroprotection

Research has revealed the potential neuroprotective benefits of RES, indicating its effectiveness in mitigating the effects of aging and neurodegenerative diseases, such as Parkinson’s and Alzheimer’s disease. In a cellular experiment on Parkinson’s disease, RES has shown promise as a therapeutic agent by significantly alleviating cytotoxicity induced by MPP+. This protective effect is mediated through the AKT/GSK-3β pathway, highlighting RES’s ability to counteract one of the key pathological processes in Parkinsons disease and prevent neuronal damage (54). Furthermore, in another *in vitro* cellular experiment, RES has been shown to effectively prevent neuronal death in immature and mature cultured cortical neurons induced by N-methyl-D-aspartate (NMDA), a factor associated with excitotoxicity in various neurological conditions. This protective effect of RES is due to its ability to regulate calcium influx triggered by NMDA receptor activation, highlighting its potential as a neuroprotective agent. Further investigation into the impact of RES on synaptic plasticity has revealed that it selectively reduces the induction of NMDA receptor-dependent long-term depression in the hippocampus without affecting long-term potentiation. This action on synaptic transmission suggests that RES can modulate neural communication in a way that may benefit cognitive function. This provides a nuanced approach to enhancing brain health and combating neurodegenerative diseases (55).

An empirical study demonstrated that administering RES orally at a dosage of 20 mg/kg increased dendrite length and spine density of pyramidal neurons in the prefrontal cortex and the CA1 and CA3 regions of the dorsal hippocampus (56). These findings indicate that RES may have the ability to safeguard and improve neural structure, which is commonly associated with age and Alzheimers disease. Besides. In an experiment lasting five months to observe cognitive performance in normal aging animals, aged rats were treated with 1.25 mg of RES daily. RES was found to increase cerebral blood flow and reduce the expression of pro-inflammatory pathways in the brain, which in turn improved cognitive performance (75). It is worth noting that the neuroprotective effects of RES are often inextricably linked to anti-inflammatory and antioxidant effects.

The neuroprotection of RES is achieved through a variety of mechanisms, and it is important to be aware of this in research. RES can activate SIRT1, which in turn maintains cell viability and delays cellular lifespan. In addition, RES reduces dopaminergic damage by decreasing the expression of the enzyme tyrosine hydroxylase. RES also regulates other enzymes involved in motor coordination. Besides, RES reduces neuronal lesions, such as atrophy of neurons and nerve fibers, and increases the α-synuclein protein degradation (76). RES can also be neuroprotective through anti-inflammatory and antioxidant effects.

## 4 Clinical application studies in RES

### 4.1 RES in disease prevention and treatment

Although laboratory and animal studies have shown numerous potential health benefits of RES, its application in clinical settings remains limited due to several primary hurdles. These include low bioavailability, the absence of large-scale clinical trials, uncertainties regarding effective dosages and optimal administration methods, and inconsistent study results. These challenges complicate the process of integrating RES into clinical practice. Nevertheless, the potential value of RES is undeniable, and it is essential to overcome these obstacles.

A study showed that daily intake of 500 mg of RES improved serum superoxide dismutase levels and total antioxidant capacity, reduced serum malondialdehyde concentrations, and enhanced the quality of life for ulcerative colitis patients (77). *Helicobacter pylori* may cause severe gastric clinical symptoms, available Chitosan nanoparticles containing RES were developed for *H. pylori* management (78). A meta-analysis investigating the impact of RES in clinical studies concluded that it significantly affects the regulation of lipid and glucose metabolism. This result highlights RES’s primary clinical usefulness in managing obesity and diabetes (79). Mucoadhesive nanoemulsions containing both RES and curcumin are available for transnasal treatment of neurodegenerative diseases (80). There is a nano-emulsion containing near-infrared dye and perfluoropolyether incorporated with RES that promises to be a versatile therapeutic strategy for cancer and other inflammatory diseases (81). Additionally, Trans-3,4,5,4′-tetramethoxystilbene, a derivative of RES, has been identified as a valuable therapeutic agent against human melanoma cells. It inhibits mitotic arrest at the pre-mid-cell division stage in A375 cells with an IC50 of 0.7 μM, demonstrating its potential for melanoma treatment (82).

### 4.2 Status and results of clinical trials

Until 2023 onwards, research on RES covers a wide range of studies that focus on health maintenance, disease prevention, and therapeutic applications. A significant portion of this research is dedicated to understanding the pharmacokinetics, distribution, metabolism, and bioavailability of various RES formulations. Clinical studies have extensively researched RES’s effects on type II diabetes and glycemic control, followed by its impact on cardiovascular diseases. Furthermore, the anti-inflammatory, antioxidant, and neuroprotective properties of RES are receiving increasing attention. However, its use in cancer treatment is not yet common in clinical settings, possibly due to difficulties in identifying accessible and alternative cancer biomarkers (83). We have compiled some of the data on RES clinical trials, as shown in Table 2.

**Table 2:** Clinical findings on resveratrol.

Perspective	Subjects	Daily dosage	Length of trial	Biomarker changes in the experimental group	Application Potential	References
Antioxidant	56 patients with active mild to moderate ulcerative colitis	500 mg placebo or RES	6 weeks	Serum superoxide dismutase and total antioxidant capacity ↑ Serum MDA ↓	Decrease disease activity and increase the quality of life	(77)
Anti-inflammatory	160 patients with periodontitis	Placebo or 500 mg, 250 mg, 125 mg RES	8 weeks	Inflammatory markers in serum and gingival crevicular fluid ↓	An efficient drug for the treatment of patients with periodontitis	(84)
Anti-diabetic	110 diabetic patients	200 mg placebo or RES	24 weeks	Plasma glucose, IR, MDA, high-sensitivity C-reactive protein, TNF-α, IL-6 miRNA-34a, miRNA-375, miRNA-21, and miRNA-192 ↓ MiRNA-126 and miRNA-132 ↑	Reduce insulin resistance and improve glycemic control	(85)
Anti-obesity	11 healthy, obese men	150 mg placebo or RES	30 days	Activated AMPK SIRT1, PGC-1α protein and citrate synthase activity ↑ Blood pressure, intrahepatic lipid content, circulating glucose, triglycerides, alanine-aminotransferase, and inflammation markers ↓	Induce metabolic changes in obese humans	(86)
Anticancer	39 adult women at increased breast cancer risk	10 or 100 mg placebo or RES	12 weeks	Total trans-resveratrol and glucuronide metabolite serum ↑ RASSF-1α methylation ↓	As a preventive measure against breast cancer	(87)
Cardiovascular Protection	17 clinical trials on blood pressure	–	–	Systolic, diastolic, mean blood pressure and pulse pressure ↓	Cardioprotective benefits	(88)
	40 post-infarction Caucasian patients	10 mg placebo or RES	3 months	Left ventricular diastolic function and endothelial function ↑ Low-density lipoprotein level ↓	Control coronary artery disease	(89)
Neuroprotection	125 postmenopausal women	150 mg placebo or RES	24 months	Overall cognitive performance, cerebrovascular function and insulin sensitivity ↑	Mitigate accelerated cognitive decline associated with aging and menopause	(90)
	119 mild to moderate Alzheimer disease patients	500 mg to 2000 mg placebo or RES	52 weeks	CSF A40 and plasma A40 levels ↓	Intervention in the central nervous system	(91)

In a randomized, double-blind, placebo-controlled trial involving diabetic patients, those who received RES supplementation showed significant improvements in blood glucose, IR, malondialdehyde, high-sensitivity C-reactive protein, IL-6, and TNF-α levels without any side effects. This indicates the potential of RES in enhancing glycemic control (85). Another study showed that 150 mg/day of RES for 30 days in obese individuals led to metabolic changes similar to those induced by energy restriction (86). The meta-analysis evaluated the effects of RES on blood pressure and found no significant impact on systolic and diastolic blood pressure in general. However, it was observed that higher doses (≥300 mg/day) significantly reduced systolic blood pressure in diabetic patients, suggesting potential cardioprotective benefits (88). RES was found to improve left ventricular function, promote endothelial function, inhibit platelet aggregation, and reduce low-density lipoprotein cholesterol levels, highlighting its potential utility in managing coronary artery disease (89). In research involving menopausal women, low-dose RES supplementation was associated with enhanced cognition, cerebrovascular function, and insulin sensitivity, potentially mitigating accelerated cognitive decline associated with aging and menopause (90). Further investigation is required to explore the implications of reducing the risk of dementia. A 52-week phase 2 trial was conducted on patients with mild to moderate AD, which found that RES and its metabolites were detectable in plasma and cerebrospinal fluid. Treatment with RES was associated with increased brain volume loss compared to the placebo (87). Further research is necessary to elucidate the changes in biomarkers in relation to RES treatment, despite its ability to cross the blood–brain barrier and have central nervous system effects. In breast cancer research, a clinical trial showed that treatment with RES over 12 weeks increased RES serum levels and affected the epigenetic pattern of the RASSF-1α gene, which is associated with breast cancer. This suggests that RES has potential as a chemopreventive agent, highlighting the need for further clinical trials to confirm these findings (91). In an eight-week trial, patients with periodontitis were orally administered low, medium and high doses of RES and a placebo. The results showed that RES not only reduced inflammatory markers in serum and gingival crevicular fluid but also decreased systemic endotoxin in periodontitis patients. This suggests that RES can inhibit systemic and localized inflammatory markers and systemic endotoxin (84).

Overall, these studies demonstrate the potential therapeutic benefits of RES for a variety of conditions. However, further research is necessary to fully comprehend its effectiveness and mechanisms of action in clinical settings.

## 5 Challenges and future research directions

### 5.1 Limitations of the current study

The challenges related to the low bioavailability and rapid metabolic processing of RES were discussed earlier. It was highlighted that only a small amount of orally consumed RES is absorbed and utilized by the body. This limitation significantly reduces the compound’s biological activity and therapeutic potential. In addition, the rapid metabolic degradation of RES in the human body leads to its quick elimination, reducing its effective concentration in the system.

There is a lack of clinical trial data on RES. Although numerous *in vitro* and animal model studies have demonstrated various potential health benefits of RES, there are comparatively few human clinical trials. And, most of these studies are limited by their small scale and short duration. Furthermore, research findings are difficult to compare and synthesize because of inconsistencies in methodology and significant differences in aspects such as dosage, method of administration, demographics, and study protocols. The fact that the optimal dose and route of administration of RES remain unclear is also an important limiting factor. Although animal research indicates potential therapeutic benefits at higher doses, it is uncertain whether these dosages are suitable for human application and how they might be safely attained via dietary or supplementary means. Notably, low concentrations of RES (0.1–1.0 μg/mL) have been found to stimulate cell proliferation, whereas higher concentrations (10.0–100.0 μg/mL) can trigger apoptosis. Therefore, further validation of effective dosages across various conditions is necessary (92).

Although RES has been associated with a range of health benefits, concerns about its safety persist. Studies have shown that RES can cause DNA damage and lead to reversible or irreversible cell cycle disruption due to its pro-oxidant effects (93). While this may explain RES’s anticancer properties, it can be harmful if the usage and dosage are not accurately controlled. Furthermore, the precise mechanisms by which RES exerts its effects are still unclear. Although multiple studies have revealed potential modes of action for RES, such as anti-inflammatory and antioxidant effects, as well as modulation of signaling pathways, the specific molecular targets and mechanisms are yet to be fully elucidated (94). For example, RES inhibits platelet aggregation. It may be related to inhibition of the p38 MAPK pathway and activation of the NO/cyclic guanosine monophosphate pathway, but it has also been suggested that platelet apoptosis is induced by high concentrations of RES, by a mechanism that has not yet been clarified (95, 96). A clearer understanding of RES’s actions could significantly enhance the ability to leverage its benefits effectively.

Regulatory and financial barriers hinder the development of nutraceuticals such as RES. Manufacturers lack the necessary infrastructure, funding, or regulatory approval. Academic institutions dominate much of the clinical research on RES, with funding coming primarily from charitable organizations or government entities. This situation has the potential to impede the achievement of major breakthroughs (83).

### 5.2 Possible directions for future research

Due to the health implications of RES, it is important to avoid subjective evaluations and broaden its potential applications beyond current research. To address the challenges mentioned earlier in RES research, it is recommended to explore innovative drug delivery mechanisms, such as nanoparticles, microemulsions, and liposomes. These mechanisms could significantly improve the bioavailability and stability of RES. By using these advanced delivery technologies, the effective concentration of RES in the body could be increased, thereby enhancing its biological efficacy.

There is a need to conduct extensive, structured, long-term human clinical trials to ascertain the precise impacts and benefits of RES on specific health conditions. The studies should examine the influence of varying doses, administration methods, and treatment regimes, as well as accounting for individual variability. Establishing and adhering to standardized guidelines for RES research—including dosage determination, administration techniques, and evaluation metrics—will mitigate discrepancies across different studies. This will facilitate a more seamless comparison and comprehensive synthesis of findings. It is essential to intensify research on the safety profile of RES, particularly focusing on the implications of long-term and high-dosage usage. Studies should include diverse demographic groups, such as children, pregnant women, and the elderly, as well as individuals with varying health statuses. Besides, the use of advanced technologies in molecular biology, genomics, and proteomics is crucial in investigating the mechanism of action and molecular targets of RES. A better understanding of its cellular and molecular mode of action could greatly inform its clinical application. Furthermore, investigating the synergistic interactions between RES and other natural compounds, conducting comprehensive studies on how RES regulates cellular functions and metabolic pathways through gene expression, and exploring its impact on the human microbiome, particularly the gut flora, represent promising research avenues. These efforts could provide crucial insights, further elucidating the multifaceted benefits of RES and paving the way for its optimized therapeutic use.

Public-private partnerships could enhance funding and policy backing for natural compound research, such as RES. And innovative research and business models may offer solutions to navigate regulatory and financial hurdles typically encountered in this field.

The application of RES in the food industry should not be underestimated. Because of the multiple health properties of RES, it is possible to develop relevant functional foods that can prevent or defend against human diseases by adding appropriate amounts of RES to foods. Furthermore, it has been demonstrated that RES has the capacity to suppress the proliferation of *Listeria monocytogenes* and *Listeria innocua* at concentrations exceeding 200 μg mL^−1^, and to impede biofilm formation (97). This reveals the potential application of RES as a food preservative.

## 6 Conclusion and prospects

RES has shown potential health benefits, including antioxidant, anti-inflammatory, anti-obesity, anti-diabetic, cardiovascular protection, and neuroprotection through various mechanisms. However, challenges such as low bioavailability, rapid metabolism, and determining the effective dose of RES limit its clinical applicability and *in vivo* efficacy. However, further research is necessary to address existing challenges and transform it into an effective therapeutic agent. With additional research, RES is expected to have a crucial part in preventing and treating a vast array of diseases, thus contributing to public health.

To address these challenges, future research should prioritize new drug delivery system development that enhance the bioavailability and *in vivo* stability of RES. Additionally, larger-scale and long-term clinical trials are necessary to validate its specific benefits to human health. Furthermore, more comprehensive studies on the mechanism of action, safety, dose-effect relationship, and synergistic effects of RES with other natural compounds are essential to fully exploit its potential medical value.

## Author contributions

XY: Writing – original draft, Writing – review & editing. YJ: Writing – review & editing. FR: Writing – review & editing.
